# Independent Prognostic Value of Single and Multiple Non-Specific 12-Lead Electrocardiographic Findings for Long-Term Cardiovascular Outcomes: A Prospective Cohort Study

**DOI:** 10.1371/journal.pone.0157563

**Published:** 2016-06-30

**Authors:** Mitsuaki Sawano, Shun Kohsaka, Tomonori Okamura, Taku Inohara, Daisuke Sugiyama, Yasuyuki Shiraishi, Makoto Watanabe, Yasuyuki Nakamura, Aya Higashiyama, Aya Kadota, Nagako Okuda, Yoshitaka Murakami, Takayoshi Ohkubo, Akira Fujiyoshi, Katsuyuki Miura, Akira Okayama, Hirotsugu Ueshima, NIPPON DATA 80/90 Research Group

**Affiliations:** 1 Department of Cardiology, Keio University Hospital, Tokyo, Japan; 2 Department of Preventive Medicine and Public Health, Keio University, Tokyo, Japan; 3 Department of Preventive Cardiology, National Cerebral and Cardiovascular Center, Suita, Japan; 4 Department of Cardiovascular Epidemiology, Kyoto Women’s University, Kyoto, Japan; 5 Department of Preventive Medicine and Epidemiologic Informatics, National Cerebral and Cardiovascular Center, Osaka, Japan; 6 Center for Epidemiologic Research in Asia, Shiga University of Medical Science, Shiga, Japan; 7 Department of Health and Nutrition, University of Human Arts and Sciences, Saitama, Japan; 8 Department of Medical Statistics, Toho University School of Medicine, Tokyo, Japan; 9 Department of Hygiene and Public Health, Teikyo University School of Medicine, Tokyo, Japan; 10 Department of Health Science, Shiga University of Medical Science, Otsu, Japan; 11 Research Institute for lifestyle-related disease prevention, Tokyo, Japan; Indiana University School of Medicine, UNITED STATES

## Abstract

**Aims:**

The long-term prognostic effect of non-specific 12-lead electrocardiogram findings is unknown. We aimed to evaluate the cumulative prognostic impact of axial, structural, and repolarization categorical abnormalities on cardiovascular death, independent from traditional risk scoring systems such as the Framingham risk score and the NIPPON DATA80 risk chart.

**Methods and Results:**

A total of 16,816 healthy men and women from two prospective, longitudinal cohort studies were evaluated. 3,794 (22.6%) individuals died during a median follow-up of 15 years (range, 2.0–24 years). Hazard ratios for cardiovascular death, all-cause death, coronary death and stroke death were calculated for the cumulative and independent axial, structural, and repolarization categorical abnormalities adjusted for the Framingham risk score and the NIPPON DATA80 risk chart. Individuals with two or more abnormal categories had a higher risk of cardiovascular death after adjustment for Framingham risk score (men: HR 4.27, 95%CI 3.35–5.45; women: HR 4.83, 95%CI 3.76–6.22) and NIPPON DATA80 risk chart (men: HR 2.39, 95%CI 1.87–3.07; women: HR 2.04, 95%CI 1.58–2.64).

**Conclusion:**

Cumulative findings of axial, structural, and repolarization abnormalities are significant predictors of long-term cardiovascular death in asymptomatic, healthy individuals independent of traditional risk stratification systems.

## Introduction

Cardiovascular disease (CVD) is a major global burden and identification of individuals at high risk of developing CVD is crucial for effective primary prevention. The risk of developing CVD varies by individual and is dependent on his or her profile; hence, clinical risk assessment scoring systems such as the Framingham risk score (FRS) or risk probability calculations using the NIPPON DATA80 risk chart (NDRC) have historically played an important role in the early detection of high-risk individuals for CVD.[1, 2] More recently, the clinical risk score has been utilized when considering medications aimed at primary prevention.[3]

In contrast, the validity of non-invasive screening tests such as the 12-lead electrocardiogram (ECG) remains controversial. Previous studies have reported the significant prognostic value of individual ECG findings such as Q-QS abnormalities, ST-T abnormalities, high R waves, atrial fibrillation, or atrial flutters and blocks.[4, 5] However, in these studies, high-risk ECG findings were investigated and thus the prognostic potential of non-specific ECG findings remains unclear. The current US Preventive Services Task Force (USPSTF) guidelines for screening asymptomatic adults with resting or exercise ECG do not recommend the use of ECG in low-risk individuals. However, these guidelines do acknowledge that current evidence is insufficient to fully assess the benefits or harm in intermediate- to high-risk individuals.[6]

The prognostic value of non-specific ECG findings may be more accurately evaluated when clustered according to ECG categories representative of the underlying electrophysiological pathology, including axial, structural, and repolarization.[7] Therefore, the present study aimed to clarify the cumulative prognostic value of axial, structural, and repolarization abnormalities of resting ECG results. Specifically, we aimed to identify its prognostic value beyond traditional risk assessment scores like the FRS and NDRC.

## Methods

### Study Population

The NIPPON DATA80 and 90 studies were 2 cohort studies of 300 randomly selected districts throughout Japan and were conducted by the National Survey on Circulatory Disorders. Participant enrollment in NIPPON DATA80 began in 1980 and NIPPON DATA90 in 1990. The specifics of these studies have been previously reported.[2, 7–13] Approval for the present study was obtained from the institutional review board of Shiga University of Medical Science. In this study, we analyzed the combined data from both the NIPPON DATA80 and 90 studies including a total of 18,929 healthy participants aged 30 years or older (10,546 from NIPPON DATA80 and 8,383 from NIPPON DATA90)(S1 Fig).

Participants in NIPPON DATA80 were followed from 1980 to 2004 and those in NIPPON DATA90 from 1990 to 2005. Cohort data included the participants’ medical histories, physical examination results, laboratory test results, standard 12-lead ECG findings, and completed self-administered questionnaire on lifestyle.

Of 18,929 participants, 2,113 were excluded for the following reasons: unavailability of a present physical address required to link to vital statistical records (n = 1388, 1104 from NIPPON DATA80 and 284 from NIPPON DATA90), missing information in the baseline survey (n = 118, 2 from NIPPON DATA80 and 116 from NIPPON DATA90), a history of known myocardial infarction or stroke (n = 392, 153 from NIPPON DATA80 and 239 from NIPPON DATA90), and specific ECG findings including a moderate or severe Q-wave abnormality (Minnesota Code, MC, 1–1, 1–2), complete atrioventricular block (MC 6–1), Wolff–Parkinson–White syndrome (MC 6–4), or atrial fibrillation or flutter (MC 8-3-1 or 8-3-2) (n = 215, 122 from NIPPON DATA80 and 93 from NIPPON DATA90). The final sample size for our analyses was 16,816 participants.

### Baseline Examinations

The baseline surveys were conducted at public health centers according to a standardized manual. Trained nurses using a standard mercury sphygmomanometer on the right arm measured blood pressure. Height and weight were measured in subjects without shoes and with light clothing. Body mass index (BMI) was calculated as weight (kg) divided by the height squared (m^2^). Blood samples were drawn and centrifuged within 60 minutes of collection and stored at −70°C until later analysis as previously described.[2]^,^ [7]^,^ [8]^,^ [10]^,^ [11]^,^ [12]

A standard, resting 12-lead ECG was recorded at the point of initiation of each cohort study, 1980 for NIPPON DATA80 participants and 1990 for NIPPON DATA90 participants. Two independent ECG examiners from the National Survey on Circulatory Disorders evaluated and coded the ECGs in accordance with Minnesota Code guidelines. When coding was discordant, a panel of epidemiologists and cardiologists assessed the data for the purposes of appropriate classification.

### ECG Abnormal Findings and Categorization

Baseline ECG findings were coded in accordance with the Minnesota Code guidelines and defined as described in Table 1. [14, 15] Individuals were divided into 3 groups according to the number of abnormal categories satisfied: none, single, or greater than two.

**Table 1:** Definition of the ECG abnormal categories.

	MC*	Definition
Axial abnormality (Left axis deviation OR Clockwise rotation)		
	MC 2–1.	QRS axis from -300 through -900 in leads I, II, III (The algebraic sum of major positive and major negative QRS waves must be zero or positive in I, negative in III, and zero or negative in II)
	MC 9-4-2.	QRS transition zone at V4 or to the left of V4 on the chest.
Structural abnormality (Left ventricular hypertrophy OR Atrial enlargement)		
	MC 3–1.	High amplitude R waves, Left: R amplitude > 26 mm in either V5 or V6, or R amplitude > 20.0 mm in any of leads I, II, III, aVF, or R amplitude > 12.0 mm in lead aVL (Measured only on second to last complete normal beat)
	MC 3–3.	High amplitude R waves, Left (optional code when 3–1 is not present): R amplitude > 15.0 mm but ≤ 20.0 mm in lead I, or R amplitude in V5 or V6, plus S amplitude in V1 > 35.0 mm. (Measured only on second to last complete normal beat)
	MC 9–3.	P-wave amplitude ≥ 2.5 mm in any of leads II, III, aVF, in a majority of beats)
Repolarization abnormality (Minor and Major ST–T changes)		
	MC 4-1-1.	STJ depression ≥ 2.0 mm and ST segment horizontal or downward sloping in the anterolateral site or the anterior site
	MC 4-1-2.	STJ depression ≥ 1.0 mm but < 2.0 mm, and ST segment horizontal or downward sloping in the anterolateral site or the anterior site
	MC 4–2.	STJ depression ≥ 0.5 mm and < 1.0 mm and ST segment horizontal or downward sloping in the anterolateral site or the anterior site,
	MC 4–3.	No STJ depression as much as 0.5 mm but ST segment downward sloping and segment or T-wave nadir ≥ 0.5 mm below P-R baseline, in the anterolateral site or the anterior site
	MC 4–4.	STJ depression ≥ 1.0 mm and ST segment upward sloping or U-shaped, in the anterolateral site or the anterior site, MC 5–1. T amplitude negative 5.0 mm or more when R amplitude is ≥ 5.0 mm
	MC 5–2.	T amplitude negative or diphasic (positive-negative or negative-positive type) with negative phase at least 1.0 mm but not as deep as 5.0 mm when R amplitude is ≥ 5.0 mm
	MC 5–3.	T amplitude zero (flat), or negative, or diphasic (negative-positive type only) with less than 1.0 mm negative phase when R amplitude is ≥ 5.0 mm, MC 5–4. T amplitude positive and T/R amplitude ratio < 1/20: R wave amplitude must be ≥ 10.0 mm) abnormalities.

*MC = Minnesota code

### CVD Risk Estimation and Stratification

The simple, office-based FRS and NDRC were calculated for each individual.[1, 2] Age, gender, BMI, systolic blood pressure, diabetes mellitus, and current smoking status were used to calculate the FRS for predicting a 10-year risk of CVD. CVD was defined as a composite endpoint of CHD (including coronary death, myocardial infarction, coronary insufficiency, and angina), cerebrovascular events (ischemic stroke, hemorrhagic stroke, and transient ischemic attack), peripheral artery disease (intermittent claudication), and heart failure. Age, gender, systolic blood pressure, total cholesterol level, diabetes mellitus, and current smoking status were used to calculate the NDRC for predicting a 10-year risk of death from all cardiovascular diseases. The underlying causes of cardiovascular death were coded according to the 9th International Classification of Disease (ICD-9) until late 1994 and the 10th International Classification of Disease (ICD-10) from early 1995 and these were 393–459 and I00-I99 respectively.[16] Individuals were divided into five risk categories; <3%, 3–7%, 7–12%, 12–20%, and > = 20% according to their FRS- and NDRC-estimated CVD and cardiovascular death risks.

### Study Endpoint

We analyzed the prognostic effect of none, single, or more than two abnormal ECG categories for the following endpoints: all-cause, cardiovascular, coronary, and cerebrovascular deaths.

### Statistical Analysis

Differences in baseline characteristics between the 3 ECG abnormal category groups were tested. Continuous variables were reported as the mean ± SD. Categorical variables were reported as percentages. P-values were based on Pearson’s chi-square tests for all categorical variables and the Student’s t-test for continuous variables. We used multivariable Cox proportional hazard regression analyses after adjustment for the five groups provided by the FRS and NDRC to calculate the hazard ratios (HRs) associated with the three ECG abnormal category groups as well as specific ECG abnormal categories: axial, structural, and repolarization. All statistical analysis was performed using Stata version 13.1 (http://www.stata.com).

## Results

### Baseline Characteristics

**Fig 1:**
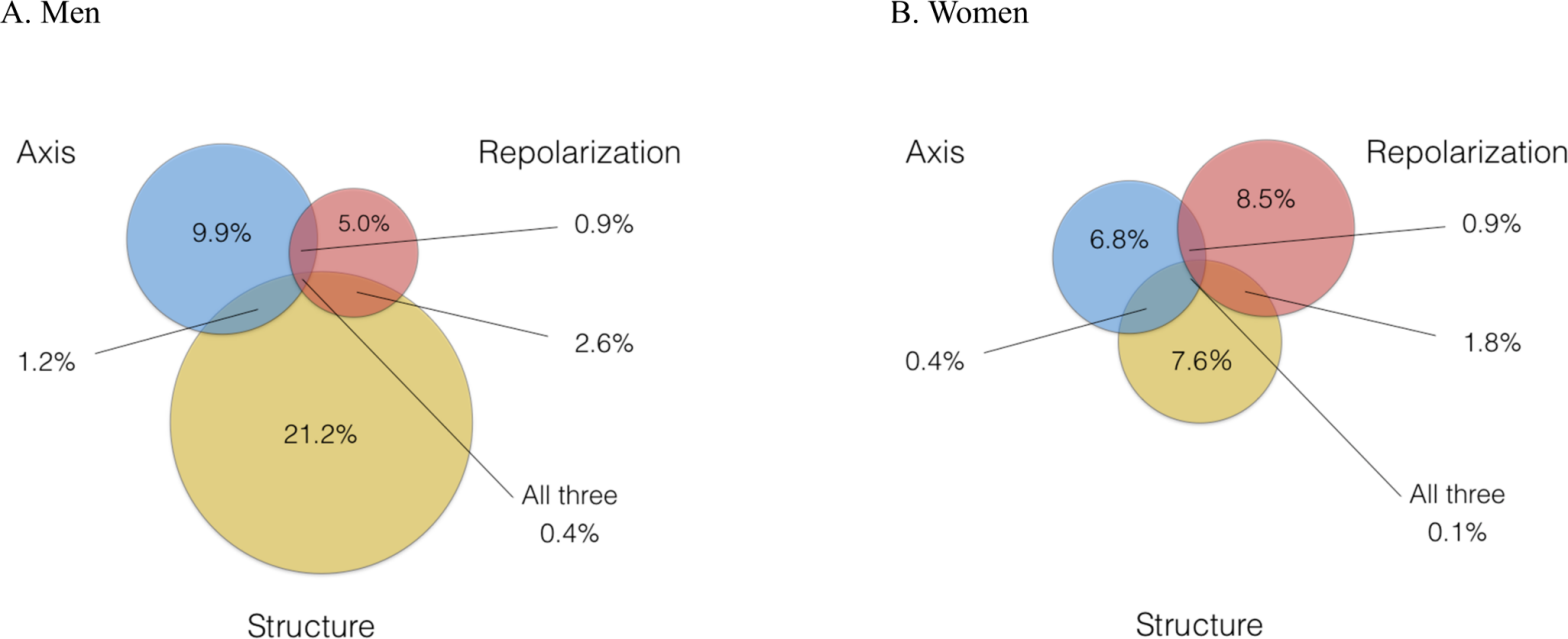
Frequency of Axial, Structural and Repolarization Abnormalities Divided by Gender. Fig 1A. Men. Fig 1B. Women.

A total of 16,816 individuals were evaluated. The mean age was 51.2 ± (standard deviation (SD)) 13.4 years and 43% were men. The follow-up duration was 300,924 person-years with a 300,924 person-years with a median follow-up of 15 years (range, 2.0–24 years). Men had a higher BMI, greater ratio of current smokers, higher systolic blood pressure, greater ratio of individuals on hypertension medication, higher total cholesterol level, and higher non-fasting blood glucose and creatinine levels (Table 2). Of the total sample, 4,203 participants (25.0%) were categorized into the following ECG abnormality groups: 3,648 (21.7%) had a single abnormality and 555 (3.3%) had greater than two abnormalities. Axial abnormalities were observed in 1362 participants (8.1%), structural abnormalities in 2,252 individuals (13.4%) and repolarization abnormalities in 1,184 participants (7.0%). When divided by gender, a higher proportion of structural abnormalities were observed in men while a higher proportion of repolarization abnormalities were observed in women (Fig A&B in Fig 1).

**Table 2:** Baseline Characteristics of the NIPPON DATA 80/90* Cohort.

	Men	Women
	7,173	9,643
Age (years)	51.1 (13.3)	51.0 (13.5)
Body mass index (kg/m2)	22.7 (2.9)	22.8 (3.3)
Current smoker (%)	4310 (60)	874 (9.0)
Systolic blood pressure (mmHg)	138 (21)	134 (21)
Antihypertensive use (%)	767 (11)	1276 (13)
Laboratory tests		
Total cholesterol (mg/dl)	191 (35)	198 (37)
Non-fasting blood glucose (mg/dl)	118 (39)	117 (35)
Creatinine (mg/dl)	1.00 (0.29)	0.79 (0.23)

* This study cohort is a combined cohort of the NIPPON DATA80 and NIPPON DATA90. Participants were followed from 1980 to 2004 in the NIPPON DATA80 cohort, and from 1990 to 2005 in the NIPPON DATA90 cohort.

Categorical values shown are n (%), unless stated otherwise. Continuous values are shown in mean (standard deviation), unless stated otherwise.

### CVD Risk Estimation and Stratification

The FRS for estimating the 10-year risk of cardiovascular events and the NDRC for estimating the 10-year risk of cardiovascular mortality was calculated for each individual. In men, a FRS median of 3.81% (Q1-3, 0.86–18.9%) (N = 7,173) and NDRC median of 0.73% (Q1-3, 0.21–3.08%) (N = 6,952; 221 men with missing blood glucose values) was observed (Fig A&B in S2 Fig). In women, a FRS median of 1.1% (Q1-3, 0.34–4.05%) (N = 9,643) and NDRC median of 0.56% (Q1-3, 0.14–2.63%) (N = 9,347; 296 women with missing blood glucose values) was observed (Fig A&B in S3 Fig). The study population was divided into five groups according to absolute risks calculated by the FRS and NDRC, and this is presented in Table 3.

**Table 3:** The Number of Individuals Accounting for the Framingham Risk Score and the NIPPON DATA80 Risk Chart.

**Men**							
		**NDRC**					
		0–3%	3–7%	7–12%	12–20%	> = 20%	Total
FRS	0–3%	2,370	363	180	126	128	3,167
	3–7%	771	130	43	34	25	1,003
	7–12%	426	87	24	18	21	576
	12–20%	391	56	34	29	20	530
	> = 20%	1,223	212	108	63	70	1,676
	Total	5,181	848	389	270	264	6,952
**Women**							
		**NDRC**					
		0–3%	3–7%	7–12%	12–20%	> = 20%	
FRS	0–3%	5,441	543	220	181	161	6,546
	3–7%	789	180	82	60	80	1191
	7–12%	357	78	39	28	26	528
	12–20%	235	79	37	24	30	405
	> = 20%	379	136	65	46	52	678

NDRC: Risk probability due to NIPPON DATA80 risk chart; FRS: Framingham risk score

### Outcomes

**Fig 2:**
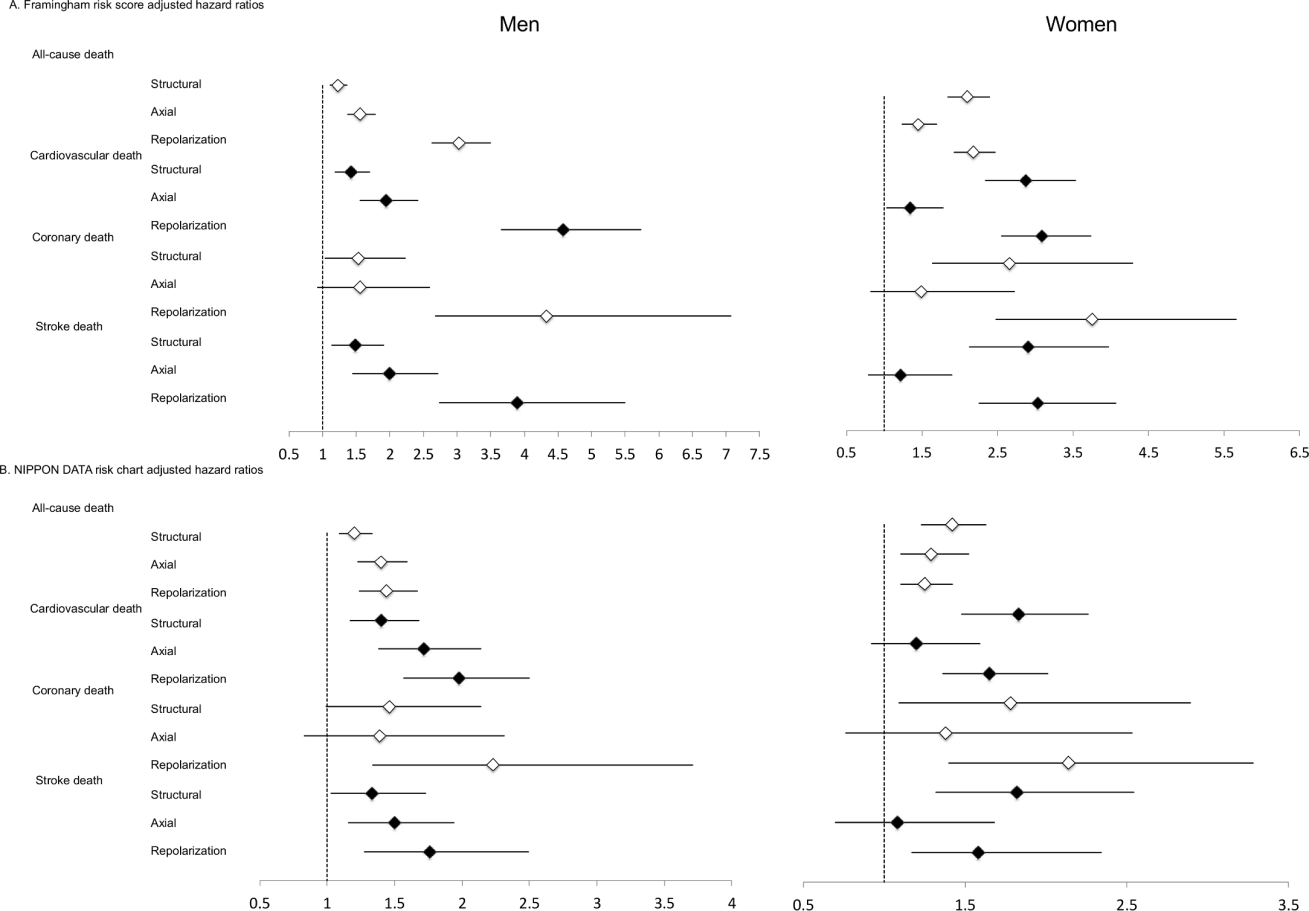
Hazard Ratios Adjusted for Framingham Risk Score and Risk Probability with NIPPON DATA80 Risk Chart.

A total of 3,794 (22.6%) individuals died during the follow-up period. Of these, 1,218 (7.2%) were cardiovascular deaths, 248 (1.5%) were coronary deaths, and 548 (3.3%) were stroke deaths. Cox proportional hazard model analysis was used to evaluate the validity of the individual ECG abnormal categories (axial, structural and repolarization) for all-cause, cardiovascular, coronary, and stroke death, and this was adjusted for FRS (Fig 2A) and NDRC (Fig 2B) (S1 Table and S2 Table). When the ECG abnormalities were assessed cumulatively, both men and women with two or more cumulative ECG abnormality categories showed significant prognostic value for all 4 endpoints (Tables 4 & 5). When categories were assessed individually, repolarization abnormalities had a consistent prognostic impact on all 4 endpoints after adjustment for FRS or NDRC in both genders while structural abnormalities was significantly associated with the 4 endpoints except when they were used to predict all-cause death and coronary death after adjustment for NDRC in men. Axial abnormalities was significantly associated with the 4 endpoints except when they were used to predict; coronary death after adjustment for FRS or NDRC in men; coronary death and stroke death after adjustment for FRS in women; and cardiovascular death, coronary death and stroke death after adjustment for NDRC in women.

**Table 4:** Impact of the Number of Abnormal ECG Categories on All-Cause and Cardiovascular Death in Men.

		All-Cause Death		Cardiovascular Death		Coronary Death		Stroke Death	
		(1,998 events)		(595 events)		(128 events)		(282 events)	
	N (%)	HR (95%CI)	*P value*	HR (96%CI)	*P value*	HR (97%CI)	*P value*	HR (98%CI)	*P* value
FRS Adjusted HRs									
No abnormality	4,896 (68)	Reference		Reference		Reference		Reference	
Single abnormality	1,996 (28)	1.26 (1.14, 1.38)	<0.001	1.26 (1.06, 1.50)	0.008	1.32 (0.91, 1.91)	0.14	1.26 (0.98, 1.62)	0.07
≥2 Abnormalities	2,84 (4)	2.59 (2.20, 3.05)	<0.001	4.27 (3.35, 5.45)	<0.001	3.63 (2.08, 6.33)	<0.001	4.17 (2.91, 5.98)	<0.001
NDRC Adjusted HRs									
No abnormality	4,703 (68)	Reference		Reference		Reference		Reference	
Single abnormality	1,970 (28)	1.22 (1.11, 1.35)	<0.001	1.22 (1.03, 1.45)	0.02	1.23 (0.85, 1.79)	0.26	1.23 (0.95, 1.57)	0.11
≥2 Abnormalities	279 (4)	1.54 (1.30, 1.81)	<0.001	2.39 (1.87, 3.07)	<0.001	2.26 (1.29, 4.00)	0.004	2.32 (1.62, 3.35)	<0.001

FRS: Framingham risk score was calculated using age, gender, body mass index, systolic blood pressure, diabetes mellitus, and current smoking. NDRC: Risk probability using the NIPPON DATA80 risk chart was calculated using age, gender, systolic blood pressure, total cholesterol level, diabetes mellitus, and current smoking. Gender was not accounted for in calculation of the FRS and the NDRC as only men were analyzed.

**Table 5:** Impact of the Number of Abnormal ECG Categories on All-Cause and Cardiovascular Death in Women.

		All-Cause Death		Cardiovascular Death		Coronary Death		Stroke Death	
		(1,796 events)		(623 events)		(120 events)		(266 events)	
	N (%)	HR (95%CI)	*P value*	HR (96%CI)	*P value*	HR (97%CI)	*P value*	HR (98%CI)	*P value*
FRS Adjusted HRs									
No abnormality	7,719 (80)	Reference		Reference		Reference		Reference	
Single abnormality	1,654 (17)	1.63 (1.46, 1.81)	<0.001	1.72 (1.43, 2.05)	<0.001	1.80 (1.21, 2.70)	0.004	1.65 (1.25, 2.17)	<0.001
≥2 Abnormalities	273 (3)	3.03 (2.53, 3.62)	<0.001	4.83 (3.76, 6.22)	<0.001	5.15 (2.94, 9.02)	<0.001	4.78 (3.25, 7.06)	<0.001
NDRC Adjusted HRs									
No abnormality	7,471 (80)	Reference		Reference		Reference		Reference	
Single abnormality	1,612 (17)	1.31 (1.18, 1.46)	<0.001	1.33 (1.11, 1.60)	0.002	1.47 (0.98, 2.20)	0.06	1.23 (0.93, 1.63)	0.152
≥2 Abnormalities	265 (3)	1.37 (1.15, 1.66)	0.001	2.04 (1.58, 2.64)	<0.001	2.32 (1.31, 4.12)	0.004	2.01 (1.35, 2.98)	0.001

FRS: Framingham risk score was calculated using age, gender, body mass index, systolic blood pressure, diabetes mellitus, and current smoking. NDRC: Risk probability using the NIPPON DATA80 risk chart was calculated using age, gender, systolic blood pressure, total cholesterol level, diabetes mellitus, and current smoking. Gender was not accounted for in calculation of the FRS and the NDRC as only men were analyzed.

## Discussion

Both screening ECG and clinical risk stratification systems (e.g. FRS and NDRC) play an important role in the early detection of high-risk individuals for atherosclerotic cardiovascular disease (ASCVD). In the present study, a total of 16,816 healthy, Japanese individuals were evaluated and we showed that the findings of the screening ECG could provide additional valuable prognostic information when findings were subcategorized by mechanism of action. Indeed, a significantly poor prognosis was observed in both men and women with either two or more ECG abnormalities or a single repolarization abnormality. Accumulation of subtle, non-significant ECG abnormalities is significant in terms of detrimental cardiovascular events and cannot be overlooked. Screening ECG may have the potential to play an additional role in the risk stratification of healthy individuals and its use should thus not be discarded.

The USPSTF guideline recommends the use of FRS to assess individual coronary heart disease risk.[6] In the present study, we found that the use of a resting ECG adds valuable prognostic information independent from that of the FRS. Thus, we recommend the use of screening ECG in CVD risk stratification. Individuals with intermediate risk calculated from clinical risk stratification systems associated with more than one ECG abnormal category may benefit from further evaluation for the prevention of fatal CVD events. Of note, the majority of individuals in this study had an FRS 10-year risk below 10%, suggesting that the overall population in our study had a low-risk profile, consistent with previous studies conducted in East Asia.[17] [18] The absolute CVD risk calculated by the NIPPON DATA80 risk chart was extremely low compared to the FRS in the present study. This result was greater than anticipated, even when taking into account the fact that the NIPPON DATA80 risk chart was designed to predict cardiovascular mortality while the FRS was designed to predict the overall incidence of cardiovascular events. It is noteworthy that the impact of cumulative ECG abnormal categories remained robust after adjustment for either the FRS or the NDRC, even in this extremely low-risk population. This may be because Japanese individuals have a greater incidence of stroke than coronary heart disease.[19]

Individually, repolarization abnormalities showed a consistent, significant prognostic value throughout the present study as previous studies have shown.[10, 20, 21] Major and minor ST-T changes are diagnosed as non-significant ST-T changes in cases where individuals do not present with any chest pain or discomfort. Although the major cause for these findings is thought to be non-ischemic in nature and includes hypertension, electrolyte disturbances, medication interference and autonomic dysfunction, there is a possibility that a considerable portion of individuals do have asymptomatic but pre-existing ischemic coronary diseases.[10] The hazard ratios for repolarization was lower in women than in men and this could be attributable to the higher frequency of major and minor ST-T changes observed in this group. Ohira et al [22] have studied the prognostic value of major and minor ST-T changes for stroke events but their results were significant only for men, despite the fact that these ECG abnormalities occurred more frequently in women. This suggests that major and minor ST-T changes tend to include normal variants in women. The exact reason for this phenomenon is unknown, but hormones, such as estrogen, as well as autonomic hyperactivity, appear to play a role.[23] Conversely, a meta-analysis conducted by Al-Zaiti et al. using sophisticated classification criteria to differentiate repolarization abnormalities suggest that these findings are particularly important when predicting sudden cardiac death in women and younger adults. Thus, further research looking into more detailed categorization of repolarization to differentiate the benign and malignant repolarization patterns is warranted. [24]

Axial and structural abnormalities showed mixed results in terms of its prognostic impact. Notably, axial abnormalities did not hold a significant impact upon predicting coronary death in both genders and stroke death in women especially after adjusting for NDRC. Similarly, structural abnormalities did not hold a significant impact upon predicting coronary death after adjusting for NDRC in men. Structural abnormalities such as left ventricular hypertrophy and atrial enlargement are frequently associated with high blood pressure. Men had a higher prevalence of hypertension and anti-hypertensive use, and the prognostic effect of these ECG abnormalities may have been minimized after adjustment for FRS and NDRC in our study compared to previous studies.[25] [26] [27] [28] [29] These results underline the importance of the cumulative effect of ECG abnormalities as the prognostic value of single ECG categorical abnormalities are limited.

An interesting finding from our study was that the prognostic effect of the cumulative ECG scores was relevant for stroke mortality as well. Unlike Western countries, Asian countries such as Japan, the Republic of Korea, the People’s Republic of China, Hong Kong, Taiwan, and the Kingdom of Thailand have a greater incidence of mortality and morbidity from stroke than from CHD.[30] Identifying risk stratification strategies for stroke events is imperative for this region, and it is possible that cumulative ECG abnormalities in fact represent the ongoing systemic risk which has the potential to result in catastrophic vascular events. A further possibility is that stroke may affect the autonomic nervous system or elevate circulatory catecholamine levels, which could result in the observed ECG abnormalities.[31]

### Limitations

There are several limitations to the present study. First, study participants were limited to Japanese ethnicity and the prognostic value of ECG abnormalities may differ among ethnicities. Second, a single 12-lead ECG was used as a baseline at study initiation and a second ECG was not performed during the follow-up period and this may have resulted in an underestimation of the risk of CVD. Third, although we have excluded the individuals with previous medical history of myocardial infarction and stroke, there is a possibility that a certain proportion of the study population in the final population that already have had an undiagnosed but pre-existing coronary artery disease that could have presented with subtle repolarization abnormalities. Since our current database was not designed to collect subsequent incidence or diagnosis of coronary artery disease, we were not able to exclude these potential coronary artery disease patients from the final analysis. Fourth, the current database recorded only Minnesota codes as the baseline assessment, thus more specific and accurate definitions such as the Romhilt-Estes point score system for left ventricular hypertrophy or P wave prolongation or P terminal force for P wave enlargement were not available. Lastly, it is not entirely clear how to act upon abnormal ECG findings. Although adding ‘categorical’ ECG abnormality classifications may provide additional benefits to the traditional scoring system, studies evaluating the effect of statins and other therapies on clinical outcomes have not yet provided clear evidence of such benefits and thus warrant further investigation.

## Conclusion

Cumulative ECG findings of axial, structural, repolarization abnormality are effective predictors of long-term cardiovascular death in asymptomatic, healthy individuals independent of traditional risk scores such as the Framingham risk score and NIPPON DATA80 risk score. Individuals with these findings require special attention in an effort to decrease the risk of future ASCVD mortality.

### Declaration

Drs. Sawano and Okamura had full access to all the data in the study and take responsibility for the integrity of the data and the accuracy of the data analysis.

## Supporting Information

S1 Fig Study Population. (TIF)

S2 Fig Men’s Distribution According to FRS and NDRC. **A**. Distribution of the Framingham Risk Score in Men **B**. Distribution of the Framingham Risk Score in Men.
(TIF)

S3 Fig Women’s Distribution According to FRS and NDRC. **A**. Distribution of the Framingham Risk Score in Women **B**. Distribution of the NIPPON DATA80 Score in Women.
(TIF)

S1 Table Impact of the Individual Abnormal ECG Category on All-Cause and Cardiovascular Death in Men. (DOCX)

S2 Table Impact of the Individual Abnormal ECG Category on All-Cause and Cardiovascular Death in Women. (DOCX)
