# Supplementary figures and images for: Independent Prognostic Value of Single and Multiple Non-Specific 12-Lead Electrocardiographic Findings for Long-Term Cardiovascular Outcomes: A Prospective Cohort Study
Source: PLoS One. 2016 Jun 30;11(6):e0157563. doi: 10.1371/journal.pone.0157563 (PMC4928789; doi:10.1371/journal.pone.0157563)

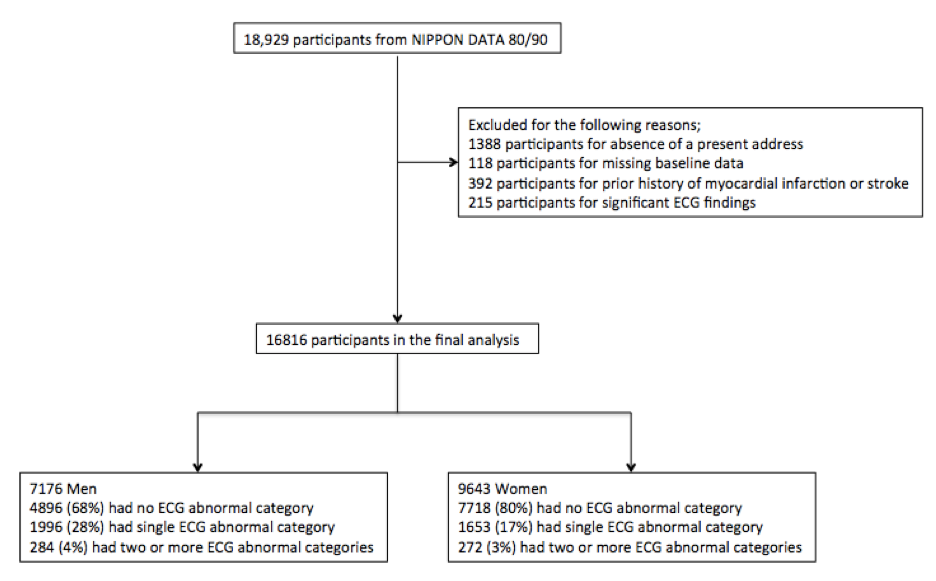

Supplement: S1 Fig — (TIF) [file pone.0157563.s001.tif]

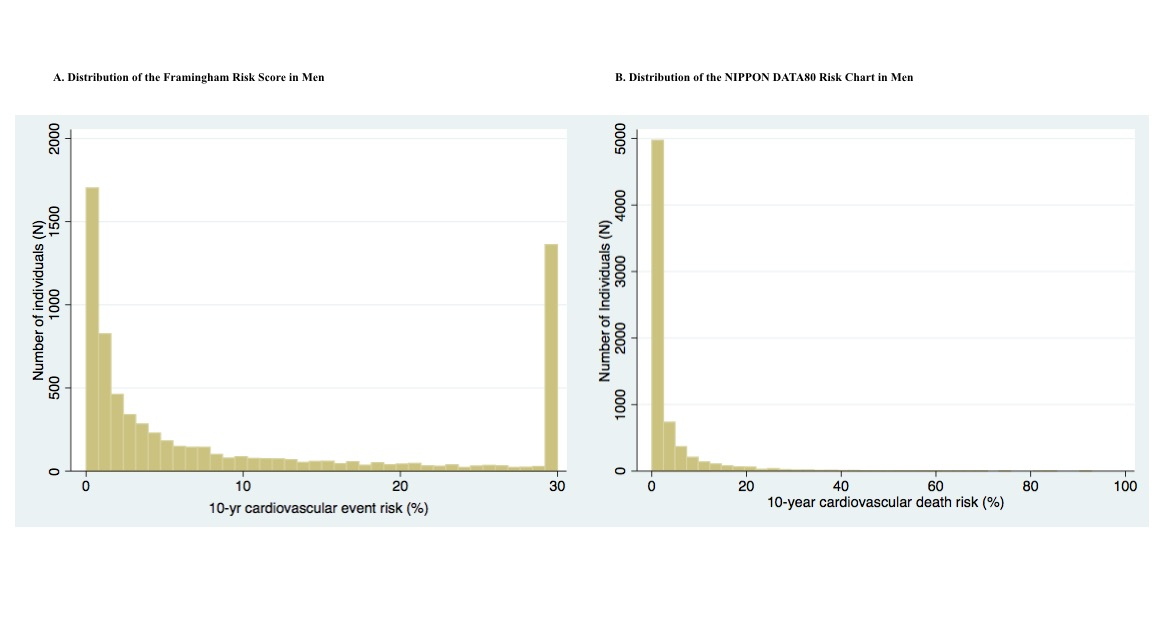

Supplement: S2 Fig — A. Distribution of the Framingham Risk Score in Men B. Distribution of the Framingham Risk Score in Men. (TIF) [file pone.0157563.s002.tif]

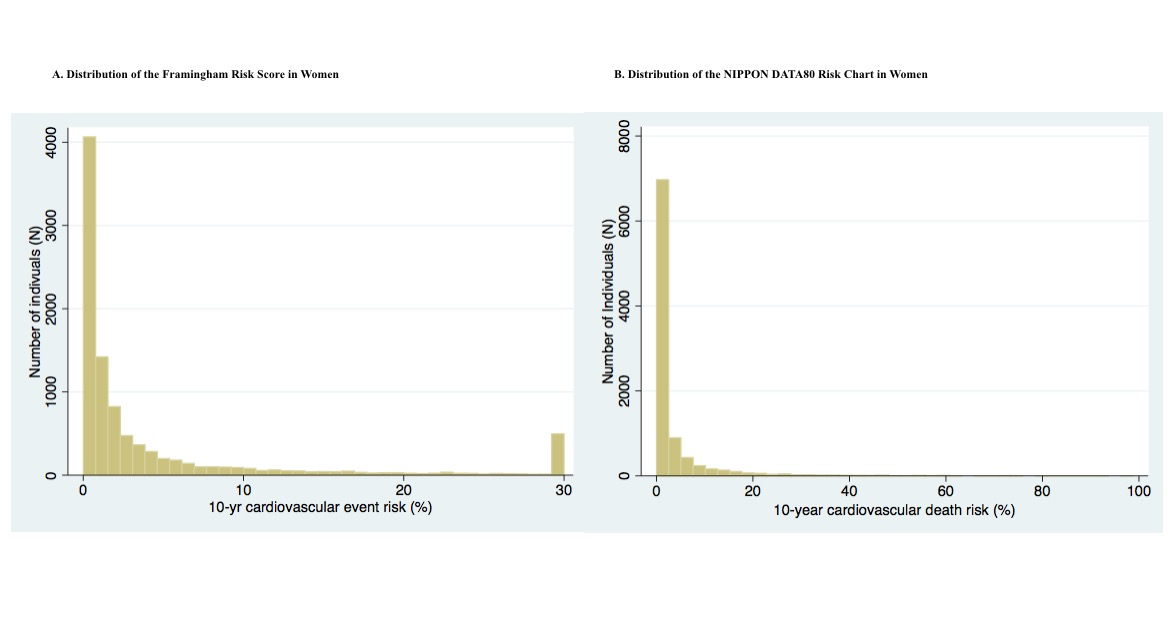

Supplement: S3 Fig — A. Distribution of the Framingham Risk Score in Women B. Distribution of the NIPPON DATA80 Score in Women. (TIF) [file pone.0157563.s003.tif]
