# Supplementary material for: Independent Prognostic Value of Single and Multiple Non-Specific 12-Lead Electrocardiographic Findings for Long-Term Cardiovascular Outcomes: A Prospective Cohort Study
Source: PLoS One. 2016 Jun 30;11(6):e0157563. doi: 10.1371/journal.pone.0157563 (PMC4928789; doi:10.1371/journal.pone.0157563)
Supplement: S1 Table — (DOCX) [file pone.0157563.s004.docx]

S1 Table. Impact of the Individual Abnormal ECG Category on All-Cause and Cardiovascular Death in Men

|  |  |  | All-Cause Death | | | | Cardiovascular Death | | | | | Coronary Death | | | | Stroke Death | | | | |
| --- | --- | --- | --- | --- | --- | --- | --- | --- | --- | --- | --- | --- | --- | --- | --- | --- | --- | --- | --- | --- |
|  |  |  | (1,998 events) | | | | (595 events) | | | | | (128 events) | | | | (282 events) | | | | |
|  |  | N (%) | HR (95%CI) | *P value* | | HR (96%CI) | | *P value* | | HR (97%CI) | | | *P value* | | HR (98%CI) | | *P* value | |  |  |
| FRS Adjusted HRs | | | | |  | |  | |  | |  | | |  | |  | |  | |  |
|  | Structural | 1,519 (21.2) | 1.23 (1.11, 1.36) | <0.001 | | 1.42 (1.19, 1.70) | | <0.001 | | 1.53 (1.04, 2.23) | | | 0.03 | | 1.48 (1.14, 1.91) | | 0.003 | |  |  |
|  | Axial | 708 (9.8) | 1.56 (1.38, 1.78) | <0.001 | | 1.95 (1.56, 2.42) | | <0.001 | | 1.56 (0.93, 2.59) | | | 0.09 | | 1.99 (1.45, 2.72) | | <0.001 | |  |  |
|  | Repolarization | 360 (5.0) | 3.03 (2.63, 3.50) | <0.001 | | 4.58 (3.66, 5.73) | | <0.001 | | 4.33 (2.65, 7.07) | | | <0.001 | | 3.89 (2.74, 5.50) | | <0.001 | |  |  |
| NDRC Adjusted HRs | | | | |  | |  | |  | |  | | |  | |  | |  | |  |
|  | Structural | 1,500 (21.2) | 1.20 (1.09, 1.33) | <0.001 | | 1.40 (1.17,1.68) | | <0.001 | | 1.46 (0.99, 2.14) | | | 0.055 | | 1.33 (1.03, 1.73) | | 0.03 | |  |  |
|  | Axial | 697 (9.8) | 1.40 (1.23, 1.59) | <0.001 | | 1.72 (1.38, 2.14) | | <0.001 | | 1.39 (0.83, 2.31) | | | 0.21 | | 1.50 (1.16, 1.94) | | 0.007 | |  |  |
|  | Repolarization | 355 (5.0) | 1.44 (1.24, 1.67) | <0.001 | | 1.98 (1.57, 2.50) | | <0.001 | | 2.23 (1.34, 3.71) | | | 0.002 | | 1.76 (1.28, 2.42) | | <0.001 | |  |  |

*FRS: Framingham risk score was calculated using age, gender, body mass index, systolic blood pressure, diabetes mellitus, and current smoking.

*NDRC: Risk probability using the NIPPON DATA80 risk chart was calculated using age, gender, systolic blood pressure, total cholesterol level, diabetes mellitus, and current smoking.

*Gender was not accounted for in calculation of the FRS and the NDRC as only men were analyzed.
