# Supplementary material for: Independent Prognostic Value of Single and Multiple Non-Specific 12-Lead Electrocardiographic Findings for Long-Term Cardiovascular Outcomes: A Prospective Cohort Study
Source: PLoS One. 2016 Jun 30;11(6):e0157563. doi: 10.1371/journal.pone.0157563 (PMC4928789; doi:10.1371/journal.pone.0157563)
Supplement: S2 Table — (DOCX) [file pone.0157563.s005.docx]

S2 Table. Impact of the Individual Abnormal ECG Category on All-Cause and Cardiovascular Death in Women

|  |  |  | All-Cause Death | | | | Cardiovascular Death | | | | | Coronary Death | | | | Stroke Death | | | | |
| --- | --- | --- | --- | --- | --- | --- | --- | --- | --- | --- | --- | --- | --- | --- | --- | --- | --- | --- | --- | --- |
|  |  |  | (1,796 events) | | | | (623 events) | | | | | (120 events) | | | | (266 events) | | | | |
|  |  | N (%) | HR (95%CI) | *P value* | | HR (96%CI) | | *P value* | | HR (97%CI) | | | *P value* | | HR (98%CI) | | *P* value | |  |  |
| FRS Adjusted HRs | | | | |  | |  | |  | |  | | |  | |  | |  | |  |
|  | Structural | 733(7.6) | 2.10 (1.84, 2.40) | <0.001 | | 2.87 (2.34, 3.53) | | <0.001 | | 2.66 (1.64, 4.29) | | | <0.001 | | 2.90 (2.13, 3.97) | | <0.001 | |  |  |
|  | Axial | 654(6.8) | 1.45 (1.24, 1.69) | <0.001 | | 1.34 (1.03, 1.78) | | 0.02 | | 1.49 (0.82, 2.72) | | | 0.19 | | 1.22 (0.79, 1.90) | | 0.367 | |  |  |
|  | Repolarization | 824(8.6) | 2.18 (1.93-2.47) | <0.001 | | 3.09 (2.55, 3.73) | | <0.001 | | 3.75 (2.48, 5.66) | | | <0.001 | | 3.03 (2.26, 4.06) | | <0.001 | |  |  |
| NDRC Adjusted HRs | | | | |  | |  | |  | |  | | |  | |  | |  | |  |
|  | Structural | 716(7.6) | 1.42 (1.23, 1.63) | <0.001 | | 1.83 (1.48, 2.26) | | <0.001 | | 1.78 (1.09, 2.89) | | | 0.02 | | 1.82 (1.32, 2.50) | | <0.001 | |  |  |
|  | Axial | 631(6.8) | 1.29 (1.10, 1.52) | <0.001 | | 1.20 (0.92, 1.59) | | 0.18 | | 1.38 (0.76, 2.53) | | | 0.28 | | 1.08 (0.70, 1.68) | | 0.74 | |  |  |
|  | Repolarization | 809(8.6) | 1.25 (1.10, 1.42) | 0.001 | | 1.65 (1.36, 2.01) | | <0.001 | | 2.14 (1.40, 3.28) | | | <0.001 | | 1.58 (1.17, 2.14) | | < 0.003 | |  |  |

*FRS: Framingham risk score was calculated using age, gender, body mass index, systolic blood pressure, diabetes mellitus, and current smoking.

*NDRC: Risk probability using the NIPPON DATA80 risk chart was calculated using age, gender, systolic blood pressure, total cholesterol level, diabetes mellitus, and current smoking.

*Gender was not accounted for in calculation of the FRS and the NDRC as only men were analyzed.*Gender was not accounted for in calculation
